# Extracellular vesicles from in vivo liver tissue accelerate recovery of liver necrosis induced by carbon tetrachloride

**DOI:** 10.1002/jev2.12133

**Published:** 2021-08-11

**Authors:** Jaemin Lee, Sae Rom Kim, Changjin Lee, Ye In Jun, Seoyoon Bae, Yae Jin Yoon, Oh Youn Kim, Yong Song Gho

**Affiliations:** ^1^ Department of Life Sciences Pohang University of Science and Technology (POSTECH) Pohang Republic of Korea; ^2^ Genome Editing Research Centre Korea Research Institute of Bioscience and Biotechnology Daejeon Republic of Korea; ^3^ Department of Medicine Yonsei University College of Medicine Seoul Republic of Korea

**Keywords:** extracellular vesicles, hepatocyte growth factor, isolation, liver failure, therapeutics, tissue engineering

## Abstract

Extracellular vesicles (EVs) are nano‐sized vesicles composed of proteolipid bilayers carrying various molecular signatures of the cells. As mediators of intercellular communications, EVs have gained great attention as new therapeutic agents in the field of nanomedicine. Therefore, many studies have explored the roles of cell‐derived EVs isolated from cultured hepatocytes or stem cells as inducer of liver proliferation and regeneration under various pathological circumstances. However, study investigating the role of EVs directly isolated from liver tissue has not been performed. Herein, to understand the pathophysiological role and to investigate the therapeutic potential of in vivo liver EVs, we isolated EVs from both normal and carbon tetrachloride (CCl_4_)‐induced damaged in vivo liver tissues. The in vivo EVs purified from liver tissues display typical features of EVs including spherical morphology, nano‐size, and enrichment of tetraspanins. Interestingly, administration of both normal and damaged liver EVs significantly accelerated the recovery of liver tissue from CCl_4_‐induced hepatic necrosis. This restorative action was through the induction of hepatocyte growth factor at the site of the injury. These results suggest that not only normal liver EVs but also damaged liver EVs play important pathophysiological roles of maintaining homeostasis after tissue damage. Our study, therefore, provides new insight into potentially developing in vivo EV‐based therapeutics for preventing and treating liver diseases.

## 1 INTRODUCTION

Liver is a pivotal major organ of the human body carrying out various important processes including detoxification, protein synthesis, and metabolites production (Fausto, 2004; Fausto et al., 2006). Liver is unique, in that it has the ability to regenerate in response to injury or damage. However, in times of severe damage caused by drugs or hepatotoxins of various origins, the regenerative capacity fails to keep up with the damage resulting in acute or chronic liver failure. Despite the ongoing challenges associated with limited donors and graft rejections (Knolle & Gerken, 2000; Lo et al., 2004; Wertheim et al., 2011), liver transplantation still remains the major and sole treatment option for treating end‐stage liver diseases (Bernal et al., 2010; Mazzaferro et al., 1996; Tripodi & Mannucci, 2011). Recently, as an alternative to organ transplantation, transplantation of primary cells such as hepatocytes (Basma et al., 2009; Bilir et al., 2000; Dhawan, 2015), stem cells (Alison et al., 2009; Duncan et al., 2009; Forbes & Newsome, 2012; Forbes & Rosenthal, 2014; Porada & Almeida‐Porada, 2010; Tosh & Strain, 2005) and hepatocytes derived from stem cells (Jang et al., 2004; Sato et al., 2005), have shown to be effective on both acute and chronic liver failures. However, the fundamental problems of cell‐based therapy regarding insufficient rate of cell survival and differentiation remain to be solved. Therefore, it is prerequisite to develop a novel approach to treat severe liver diseases.

Extracellular vesicles (EVs) such as exosomes and microvesicles produced from cells mediate various intercellular communications. EVs are nano‐sized structures composed of proteolipid bilayer enclosing numerous proteins, genetic molecules, and metabolites (Choi et al., 2013, 2015). Increasing studies on EVs have revealed the roles of EVs as important modulators in maintaining the homeostasis of healthy individuals as well as in disease pathology in patients (Al‐Nedawi et al., 2008; Antonyak et al., 2011; Thery et al., 2009; Valadi et al., 2007). EVs have gained attention as new therapeutic and biomarker candidates for several diseases, especially in cancer‐diagnosis (Fais et al., 2016; Lee et al., 2012; Lin et al., 2015; Samir et al., 2013; Skog et al., 2008). For liver diseases, cell‐based therapies including stem cells and primary hepatocytes have shown to be effective on various liver diseases. EVs secreted from liver cells are being considered as an advanced substitute to replace cell‐based approaches. Briefly, stem cell derived EVs have been shown to have therapeutic effects on acute and chronic liver disease by regulating signalling molecules involved in hepatocytes proliferation (Bruno et al., 2020; Bruno et al., 2020; Calleri et al., 2021; Dong et al., 2020; Fonsato et al., 2012; Herrera et al., 2010; Hyun et al., 2015; Li et al., 2013; Li et al., 2020; Moran & Cubero, 2018; Nong et al., 2016; Parekkadan et al., 2007; Szabo & Momen‐Heravi, 2017; Tan et al., 2014; van Poll et al., 2008; Xagorari et al., 2013). In addition, EVs derived from mouse hepatocytes alleviated the hepatic injuries in experimental murine models of hepatic ischemia/reperfusion and partial hepatectomy (Nojima et al., 2016). These previous studies so far have explored the roles of cell‐derived EVs isolated from cultured hepatocytes or stem cells as inducer of liver proliferation and regeneration under various pathological circumstances and no study has investigated the role of EVs isolated directly from in vivo liver. Therefore, in order to understand the pathophysiological role of in vivo liver EVs and to explore their therapeutic potential, further study using EVs directly isolated from liver tissue is needed.

In this regard, we here isolated EVs directly from perfused fresh liver tissues. Next, we characterized the physicochemical properties of EVs such as morphology, density, size, and protein composition. In addition, we investigated the effect of in vivo liver EVs by intravenously administrating EVs after inducing necrotic liver damage via carbon tetrachloride (CCl_4_) in murine models (Link et al., 1984; Rahman & Hodgson, 2000; Recknagel et al., 1989). Moreover, we performed time‐course experiments to trace the changes in the severity of CCl_4_‐induced hepatic damages with or without subsequent in vivo liver EV treatment. Then, we analysed the blood markers to evaluate liver damages and histologically examined the molecules associated with liver necrosis to determine the therapeutic efficacy of exogenous in vivo liver EVs. Furthermore, we compared the effect of liver tissue recovery after administration of EVs isolated from either normal or CCl_4_‐induced damaged in vivo liver tissues.

## 2 MATERIALS AND METHODS

### 2.1 Animals

C57BL/6 mice (6‐week old, male) were purchased from Jackson Laboratories and bred in specific pathogen‐free facility in POSTECH. All animal experiments were approved by the Institutional Animal Care and Use Committee at POSTECH, Pohang, Republic of Korea (approval number: POSTECH‐2016‐0047).

### 2.2 Purification of EVs from the liver tissues

We isolated in vivo EVs from liver tissues as described previously with some modification (Crescitelli et al., 2021). Livers were harvested and sliced into pieces (> 5 × 5 × 5 mm) with a razor blade. Dissected livers were incubated in serum‐free Dulbecco's Modified Eagle Medium supplemented with 1% Antibiotic‐Antimycotic (Invitrogen, Carlsbad, CA, USA) for 30 min with mild agitation at 37°C. To remove remaining cells and debris, liver perfusate, conditioned medium was centrifuged at 500 *g* for 10 min and then centrifuged at 3,000 *g* for 20 min, twice. The supernatant was pelleted at 100,000 *g* for 2 h at 4°C and the pellet was resuspended with 4.8 ml of 30% iodixanol (Axis‐Shield PoC AS, Oslo, Norway) in homogenization medium (0.25 M Sucrose, 20 mM HEPES, 150 mM NaCl, pH 7.4). The sample was placed on the bottom of an ultracentrifuge tube and was overlaid with 3.0 ml of 20% and 2.5 ml of 5% iodixanol in homogenization medium. After buoyant density gradient ultracentrifugation at 200,000 *g* for 2 h, 10 fractions of equal volume (1.0 ml) were collected from the top to the bottom of the gradient. The third fraction from the top was further diluted with HEPES‐buffered saline (25 mM HEPES, 140 mM NaCl, pH 7.2), and ultracentrifugated at 100,000 *g* for 2 h at 4°C. The pellets, in vivo liver EVs, were resuspended in HEPES‐buffered saline and the protein concentration was measured with Bradford dye assay (Bio‐Rad Laboratories, Hercules, CA, USA).

### 2.3 Transmission electron microscopy (TEM)

The purified in vivo liver EVs were placed on copper grids (Electron Microscopy Sciences, Fort Washington, PA, USA). After in vivo liver EVs were absorbed onto the grid for 30 min, the grids were washed with droplets of deionized water and negatively stained with 2% uranyl acetate (Ted Pella, Redding, CA, USA). Electron micrographs were obtained from JEM 1011 microscope (JEOL, Tokyo, Japan).

### 2.4 Measurement of EV size distribution and zeta potential

The size distribution and zeta potential of *in vivo* liver EVs were measured with Zetasizer Nano ZS (Malvern Instrument Ltd., Malvern, UK). The size distribution was determined by an infra‐red light (wavelength = 633 nm) and the zeta potential was measured in the automatic mode by detecting the electrophoretic mobility resulting from laser Doppler velocimetry.

### 2.5 Nanoparticle tracking analysis

The particle concentration of in vivo liver EVs were determined using a Nanosight LM10‐HS system (Nanosight Ltd., Amesbury, UK). Samples were injected into the chamber and were visualized using a 405 nm laser. The recorded images were analysed using the nanoparticle tracking analysis software (version 2.3).

### 2.6 Western blotting

Liver lysates (10 μg in total protein) and in vivo liver EVs (1 μg in total protein) were loaded onto SDS‐PAGE gels, and then transferred to PVDF membranes (Millipore, Bedford, MA, USA). The membrane was blocked and incubated with horseradish peroxidase‐conjugated secondary antibodies, goat anti‐rat IgG, goat anti‐hamster IgG, goat anti‐mouse IgG, and goat anti‐rabbit IgG (Santa Cruz Biotechnology, Santa Cruz, CA, USA). The immunoreactive bands were visualized using enhanced chemiluminescence substrate (Thermo Scientific, Hudson, NH, USA). Rat anti‐CD9, mouse anti‐CD81 and mouse anti‐GM130 antibodies were from BD Biosciences (BD Biosciences, San Jose, CA, USA). Rabbit anti‐histone H2B antibody was purchased from Upstate Biotechnology (Lake Placid, NY, USA).

### 2.7 CCl_4_‐induced liver injury and specimen preparation

CCl_4_ (Sigma) was prepared as a 50% (vol/vol) solution in corn oil (Sigma), and a single dose of 2 ml/kg of body weight was administered by intraperitoneal injection (*n* = 4–8) to induce hepatic injury in mice. The same volume of corn oil was used as control (*n* = 4–8) (Yoon et al., 2013). CCl_4_ and in vivo liver EVs were administered as described in Scheme. Briefly, in vivo liver EVs (5 μg in total proteins) were intravenously administered 3 h after CCl_4_ injection. Then, the same amount of in vivo liver EVs were given three times more at 24 h intervals. Mice were sacrificed 1, 2, 3, 4, and 7‐day after CCl_4_ administration. The blood was collected by eye bleeding, and serum was obtained by centrifugation at 500 *g* for 10 min and 1,500 *g* for 10 min at 4°C. The liver was harvested after whole body perfusion, and was immediately frozen by liquid nitrogen, and stored at ‐80°C.

### 2.8 Measurement of serum enzyme activity

The activity of serum alanine aminotransferase (ALT), aspartate aminotransferase (AST) and lactate dehydrogenase (LDH) were measured using an auto chemistry analyser BS‐380 (Mindray, Shenzhen, China).

### 2.9 Histological analysis and immunohistochemistry

The harvested livers were immediately fixed with 4% paraformaldehyde, embedded in paraffin, sectioned at 4‐μm thickness, and deparaffinized at 60°C oven. For liver histology, the deparaffinized liver sections were stained with haematoxylin and eosin. For immunohistochemistry, the deparaffinized liver sections were performed heat‐induced target antigen retrieval and treated with 0.3% H_2_O_2_ solution (Sigma) for blocking of endogenous peroxidase onto the liver sections. After washing, the sections were additionally blocked with protein block serum‐free blocking solution (DAKO, Glostrup, Denmark) and incubated with antibody against proliferating cell nuclear antigen (PCNA, Abcam Inc.). Then, these sections were applied the horseradish peroxidase‐conjugated secondary antibody (Santa Cruz Biotechnology) and incubated with 3,3′‐diaminobenzidine chromogen (Sigma) until the colour change of antibody to brown. All images were acquired using an Olympus BX51 light microscope (Olympus, Tokyo, Japan). Randomly captured images from four to eight independent liver sections were quantified the necrotic area and the number of PCNA‐positive cells using ImageJ software (National Institute of Mental Health, Bethesda, MD, USA; http://rsb.info.nih.gov/ij/).

### 2.10 Immunofluorescence

After the deparaffination of liver sections, heat‐induced target antigen retrieval was performed and the sections were blocked with protein block serum‐free blocking solution (DAKO, Glostrup, Denmark). Then, antibodies against active caspase‐3 (BD Biosciences), mouse α‐smooth muscle actin, and mouse F4/80 (Abcam Inc.), and mouse hepatocyte growth factor (HGF, Santa Cruz Biotechnology) were treated to the sections and incubated with Alexa 488‐conjugated donkey anti‐goat IgG, Alexa 555‐conjugated donkey anti‐rabbit IgG or Alexa 647‐conjugated donkey anti‐mouse IgG secondary antibody (Molecular Probes, Eugene, OR, USA). The nuclei were counter‐stained with Hoechst 33258 (Sigma) in the sections. All images were acquired using a Carl Zeiss LSM 700 light microscope (Carl Zeiss, Oberkochen, Germany).

### 2.11 Statistical analysis

GraphPad Prism software (Version 5) was used for statistical analysis. All values were represented as means ± standard deviation. *P* values were calculated from one way‐ANOVA. *P* values < 0.05 were considered statistically significant.

## 3 RESULTS

### 3.1 Isolation and characterization of in vivo liver EVs

**FIGURE 1:**
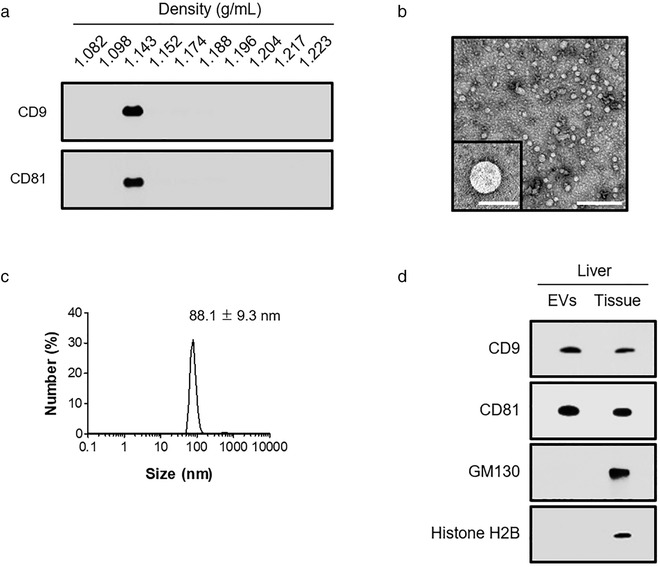
Preparation of liver tissue derived EVs. (a). Western blotting analysis of EV markers, CD9 and CD81, in fractions obtained from the iodixanol density gradients of in vivo liver EVs. (b). Representative TEM image of in vivo liver EVs. Scale bar: 500 nm; inset, 100 nm. (c). Size distribution of in vivo liver EVs measured by dynamic light scattering (*n* = 3). (d). The purified in vivo liver EVs (1 μg of total protein) and liver lysates (10 μg of total protein) were loaded to detect the EV markers, CD9 and CD81, and non‐EV markers, GM130 and Histone H2B via western blotting analysis

To isolate relatively pure *in vivo* liver EVs, mice were perfused prior to the dissection of the liver to minimize the contamination of circulating blood EVs (Crescitelli et al., 2021). The liver was dissected into relatively large pieces with each piece having a larger surface area. The dissected pieces were pre‐washed with an isotonic solution to decontaminate any damaged cellular organelles or fragments of cells produced while cutting the liver tissues. Then, EVs were passively extracted in serum‐free culture media and subsequently collected by ultracentrifugation. To increase the purity of the EV preparation, we further isolated the EVs through iodixanol buoyant density gradient ultracentrifugation. White substances were detected on the fraction with a density of 1.143 g/ml, which was exclusively positive to EV markers, CD9 and CD81, compared to other density fractions (Figures 1a and S1a). Regarding EV productivity, this procedure yielded 136.7 (± 28.9) μg of purified in vivo liver EVs which corresponds to 0.77 (± 0.16)  × 10^11^ particles from 1 g of liver tissues. We then analysed and characterized the physicochemical features of the purified in vivo liver EVs. Morphometric analysis using TEM showed that in vivo liver EVs are lipid bi‐layered spherical structures with 84.4 ± 13.5 nm in average diameter (Figure 1b). Concordantly, dynamic light scattering analysis (DLS) showed that the diameter of major population within the in vivo liver EV preparation is 88.1 ± 9.3 nm (Figure 1c). In addition, DLS measurement revealed that the average value of zeta potential of in vivo liver EVs is – 9.4 ± 0.9 mV, indicating that in vivo liver EVs are weakly anionic under physiological condition. Moreover, we found that in vivo liver EVs were highly enriched with typical EV markers, CD9 and CD81, but undetectable for markers of intracellular organelles, GM130 for Golgi, and histone H2B for nuclei via comparative western blot analysis with 10 times higher protein quantity of liver tissue homogenates (Figures 1d and S1b). Collectively, we quantified and performed characterization experiments of in vivo liver EVs including their molecular shape, size distribution, zeta potential, and composition of EV marker proteins and revealed that in vivo liver EVs share similar physicochemical characteristics with typical EVs isolated from in vitro cell conditioned medium (Thery et al., 2018).

### 3.2 In vivo liver EVs attenuate progression of hepatic injury induced by CCl_4_ treatment

**FIGURE 2:**
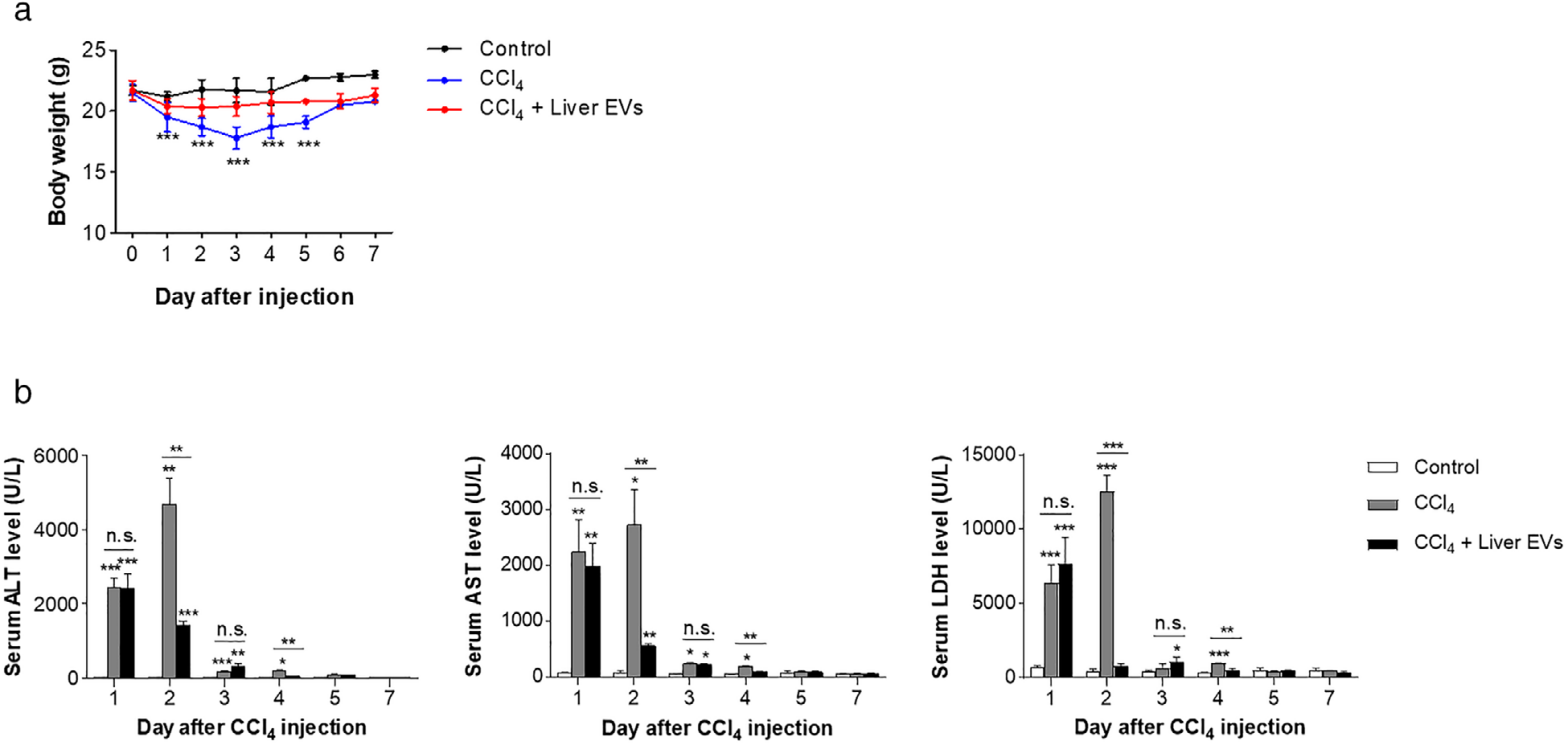
Attenuation of the progression of CCl4‐induced acute hepatotoxicity by in vivo liver EVs. (a). Body weight examined after CCl4 injection. (b). Serum levels of key enzymes, ALT, AST and LDH measured after CCl4 injection. Data are presented as the mean ± SD. n.s., not significant; * P < 0.05; ** P < 0.01; *** P < 0.001

**SCHEME 1:**
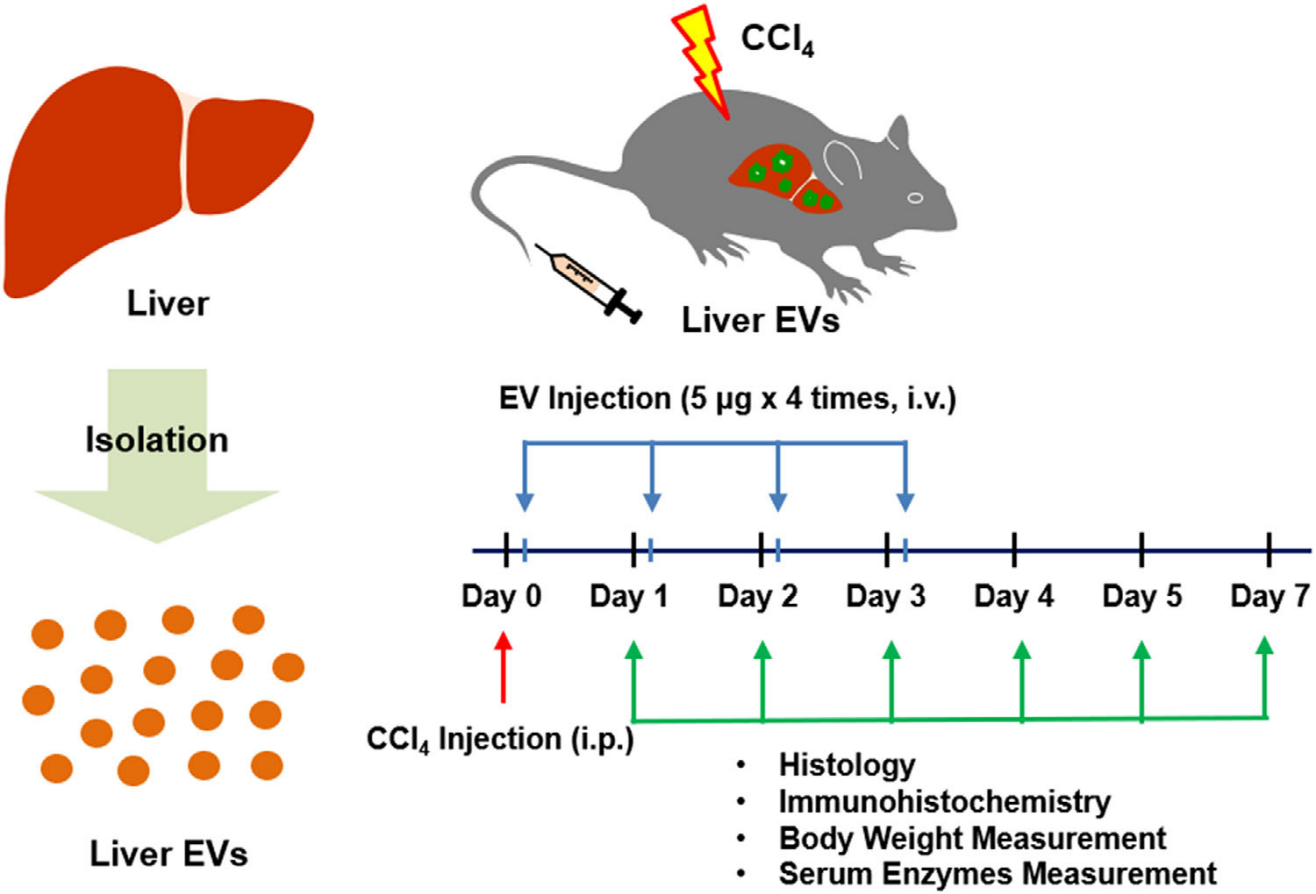
Diagram of experimental procedures in this study. We isolated EVs from freshly dissected liver tissue and subsequently injected in vivo liver EVs to CCl4‐induced acute liver injury mice model. i.p., intraperitoneal; i.v., intravenous

We hypothesized that the residential EVs within the liver tissues could contribute to maintaining the integrity of the liver and its regenerative ability. Therefore, we examined the effect of in vivo liver EVs on acute hepatic injury induced by CCl_4_, a well‐known hepatotoxic chemical causing hepatic centrilobular necrosis in rodents (Moran & Cubero, 2018). Mice intraperitoneally injected with a single dose (2 mg/kg) of CCl_4_ showed spontaneous reduction in the body weights for 3 days of post‐injection then gradually recovered to normal levels 1 week after the injection (Figure 2a). For group receiving both CCl_4_ and in vivo liver EVs, the initial dose (5 μg of total protein per animal) of in vivo liver EVs was intravenously administrated 3 h after the CCl_4_ injection, followed by three additional doses with 24‐h intervals (as shown in Scheme 1 and Materials and Methods). Interestingly, no significant change in the body weight was observed throughout the experimental period in the group receiving both CCl_4_ and in vivo liver EVs, suggesting the systemic protective effect of in vivo liver EVs on CCl_4_ toxicity. It is well known that CCl_4_‐induced hepatic necrosis and apoptosis result in the elevation of liver cytosolic enzymes in blood including ALT and AST, as well as the tissue damage marker, LDH (Kim et al., 2008). We therefore measured the activities of these enzymes in the serum collected from mice. The blood levels of these enzymes were dramatically increased and peaked at day 2 of post‐CCl_4_ treatment in the group receiving CCl_4_ alone (Figure 2b), which is concordant with the previous studies (Xie et al., 2015). With time, the enzyme levels subsided to the levels similar to that of the control group not treated with CCl_4_. In contrast, the group receiving both CCl_4_ and in vivo liver EVs exhibited significant reductions in the serum levels of these enzymes specifically at day 2 of post‐CCl_4_ treatment, implying early recovery of the liver damages upon subsequent treatment of in vivo liver EVs. Collectively, these results suggest that the treatment of in vivo liver EVs after CCl_4_ administration significantly lessen the progression of CCl_4_‐induced hepatotoxicity preventing further damages of liver.

### 3.3 In vivo liver EVs attenuate hepatic necrosis and apoptosis

**FIGURE 3:**
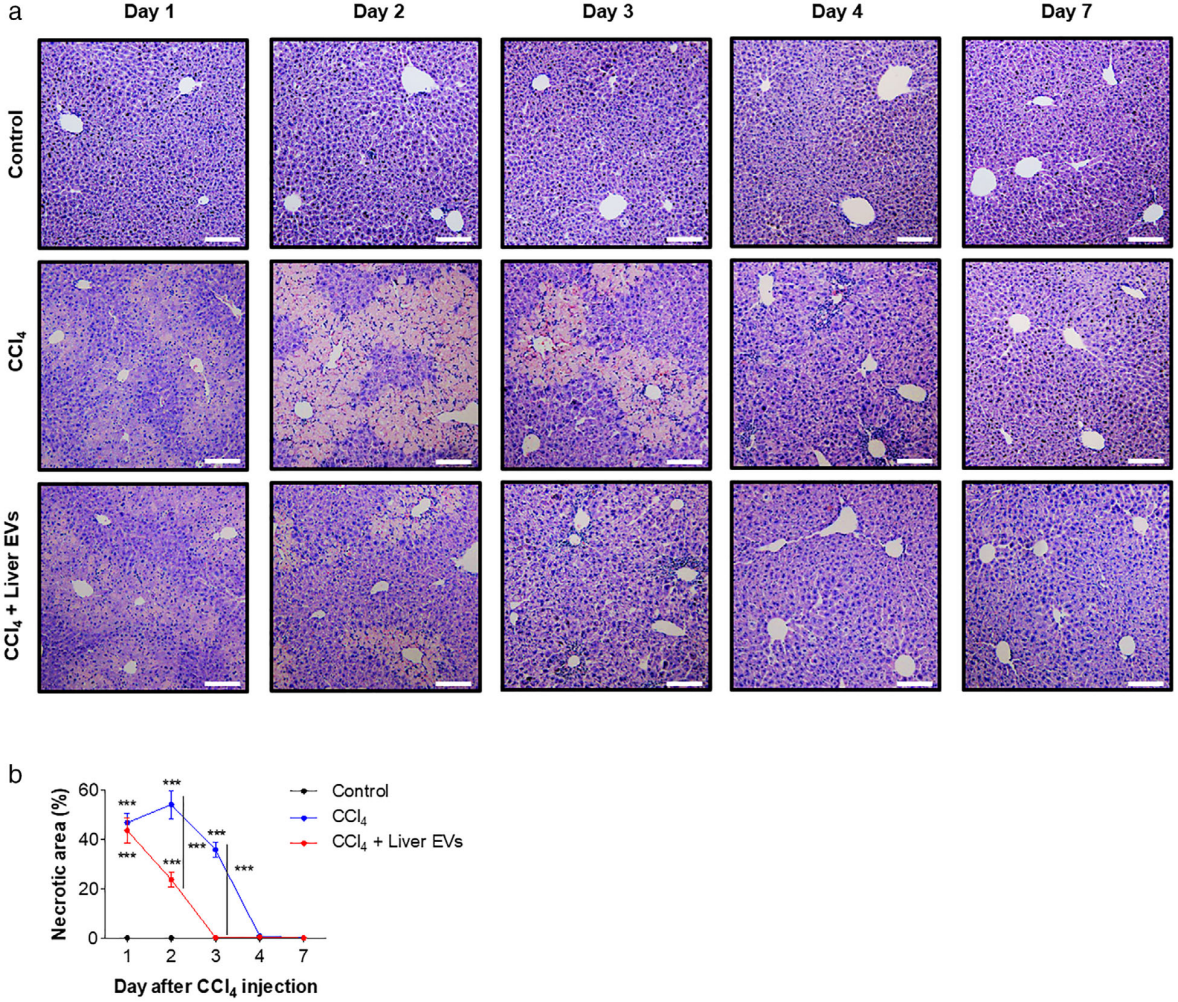
Accelerated recovery of hepatic necrosis on damaged liver tissue by in vivo liver EVs. (a). Haematoxylin and eosin staining of the mice liver sections at various time points after CCl4 injection. (b). The necrotic areas were calculated in 10 randomly selected fields per section. Scale bars: 100 μm. Data are presented as the mean ± SD. *** P < 0.001

**FIGURE 4:**
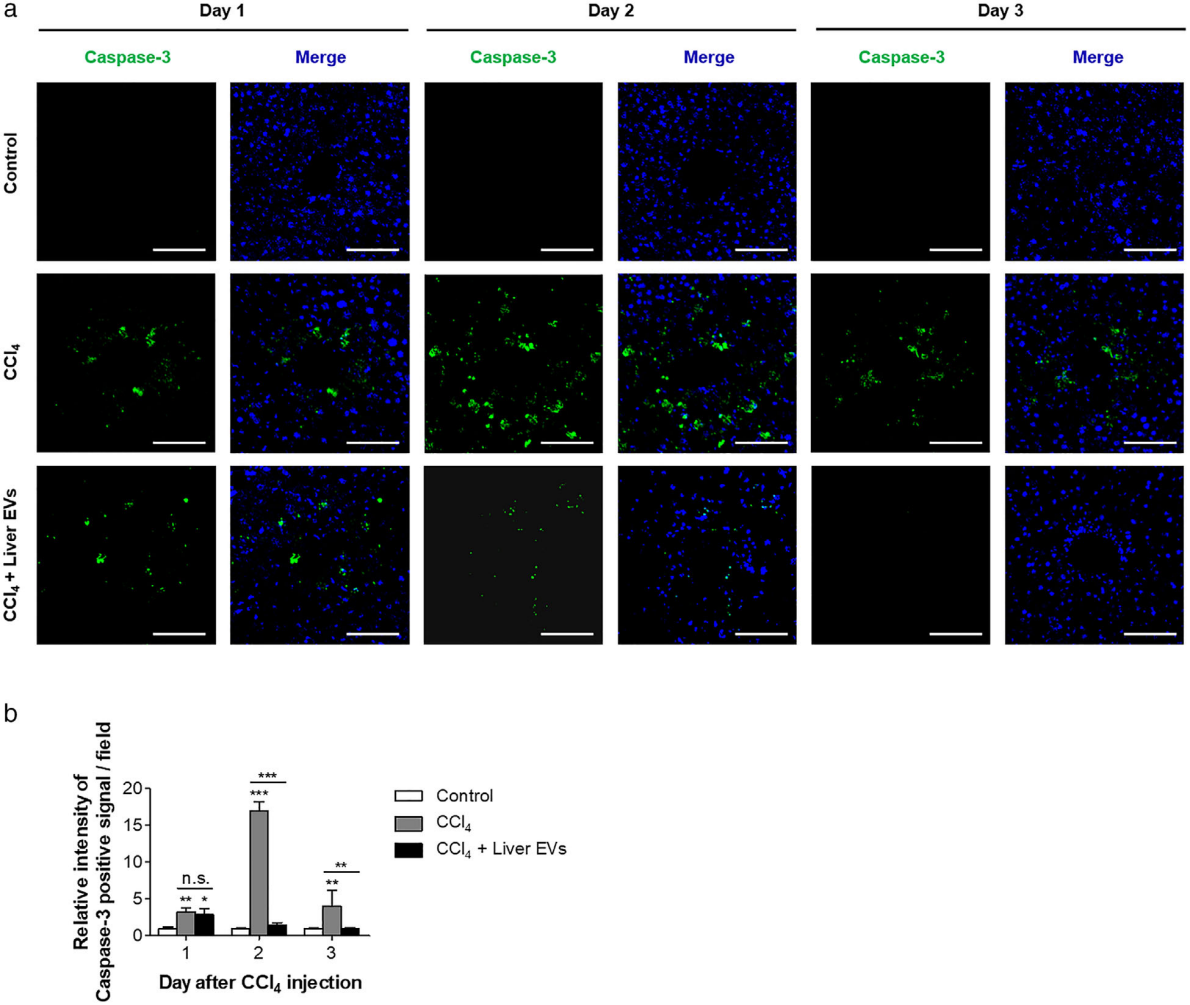
Deactivation of apoptotic pathway on damaged liver tissue by in vivo liver EVs. (a). The liver sections were stained with apoptotic marker (anti‐active caspase‐3 antibody; green), and nucleus (Hoechst; blue), and detected using a confocal microscope. (b). The relative intensities of signals were calculated in 10 randomly selected fields per image. Scale bars: 50 μm. Data are presented as the mean ± SD. n.s., not significant; * P < 0.05; ** P < 0.01; *** P < 0.001

Next, we examined liver tissue histology samples stained with haematoxylin and eosin to investigate the effect of in vivo liver EVs on the recovery of liver tissue damage by CCl_4_ injection. In the group treated with CCl_4_ alone, we found that intraperitoneal injection of CCl_4_ resulted in a significant increase of necrotic area around the central veins in the liver, which peaked at day 2 of post‐CCl_4_ treatment (Figure 3). The area replenished and recovered to normal state after two additional days. Interestingly, in the group treated with in vivo liver EVs after CCl_4_ treatment, the area of hepatic necrosis at day 2 of post‐CCl_4_ treatment was significantly less compared to that of the group not treated with in vivo liver EVs. Moreover, the group treated with in vivo liver EVs showed faster recovery of the necrotic area than the group not treated with in vivo liver EVs. It is known that CCl_4_‐induced hepatic damages involve apoptotic pathway triggered by oxidative stress and free radicals (Nojima et al., 2016). Therefore, we measured the tissue level of active caspase‐3, a marker of apoptosis, to determine if in vivo liver EVs suppress apoptotic pathway induced by CCl_4_ toxicity (Figure 4). The group not receiving in vivo liver EVs showed increased level of active caspase‐3 around the central vein area reaching its maximum at day 2 of post‐CCl_4_ treatment (Shi et al., 1998). However, the group administered with in vivo liver EVs after CCl_4_ treatment showed a significant decrease in caspase‐3 signals at day 2 and was completely free of active caspase‐3 signals at day 3 of post‐CCl_4_ treatment although the initial degree of active caspase‐3 induced by CCl_4_ at day 1 of post‐CCl_4_ treatment was similar regardless of in vivo liver EV treatment. These results suggest that in vivo liver EVs play roles on deactivating apoptotic pathway induced by CCl_4_ treatment thereby accelerating the recovery of hepatic damage.

### 3.4 In vivo liver EVs promote hepatocyte proliferation

**FIGURE 5:**
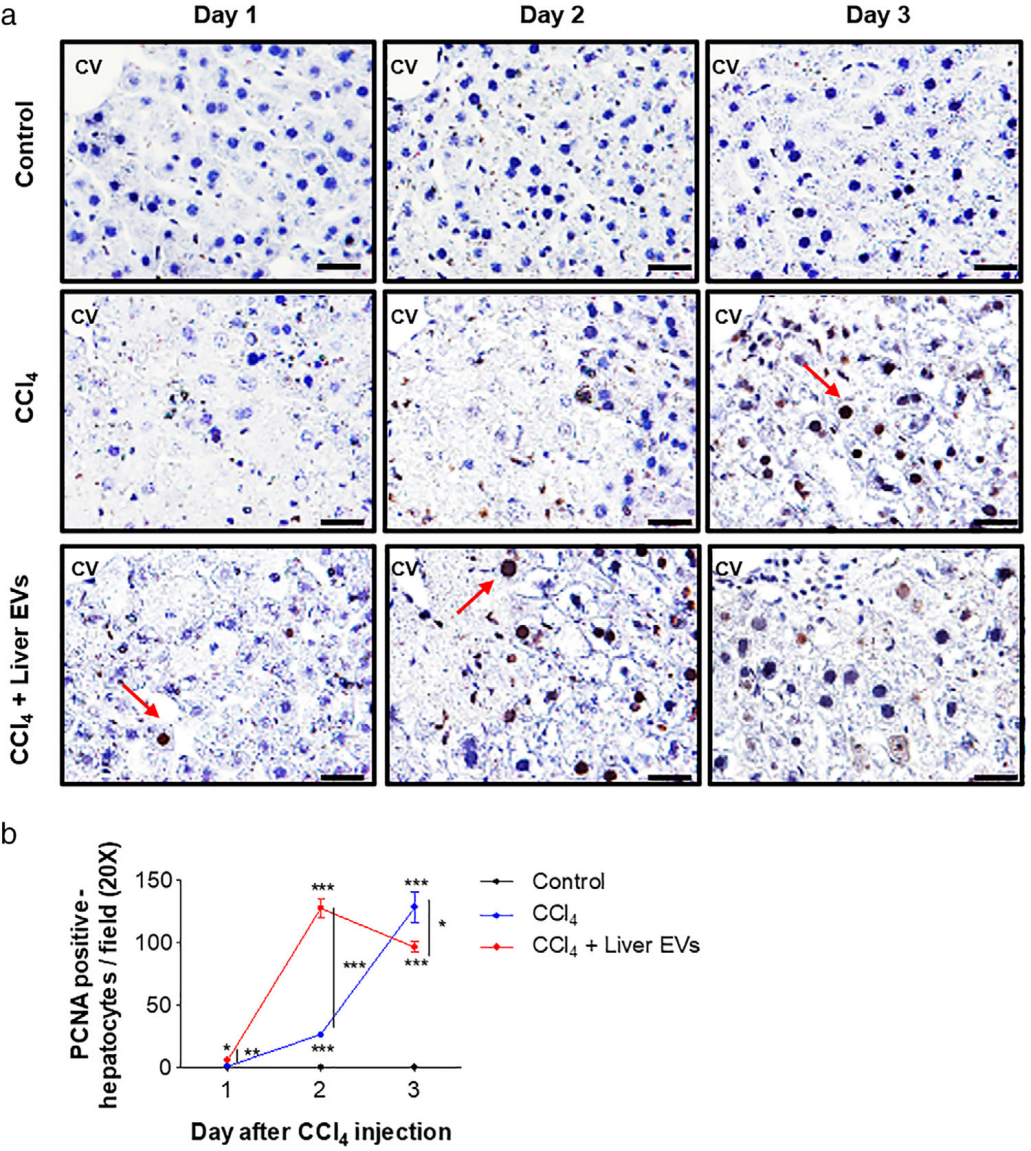
Induction of hepatocyte proliferation on damaged area by in vivo liver EVs. (a). Proliferation of hepatocyte was evaluated by the PCNA immunohistochemistry. CV: central vein. (b). The PCNA positive cells (red arrow) were calculated in 10 randomly selected fields per section. Scale bars: 25 μm. Data are presented as the mean ± SD. * P < 0.05; ** P < 0.01; *** P < 0.001

During the regenerative phase of a healthy adult liver, the cells rapidly enter the cell cycle and proliferate to replace the damaged liver tissues but rarely proliferate in normal condition. This process is mainly controlled by non‐parenchymal cells in the liver tissue including hepatic stellate cells (HSCs) and Kupffer cells (Fausto, 2004; Fausto et al., 2006; Fujiyoshi & Ozaki, 2011). Since EVs secreted from these types of cells also comprise in vivo liver EVs, we examined if in vivo liver EVs are capable of inducing hepatocyte proliferation by measuring PCNA. As shown in Figure 5, the number of PCNA‐positive hepatocytes are significantly increased in the group receiving both CCl_4_ and in vivo liver EVs at day 2 of CCl_4_ post‐injection compared with the groups not receiving in vivo liver EVs. These results support that in vivo liver EVs comprising various EVs secreted from parenchymal and non‐parenchymal cells trigger mechanisms which promote the recovery of damaged tissues by inducing hepatocytes proliferation.

### 3.5 In vivo liver EVs induce HGF

**FIGURE 6:**
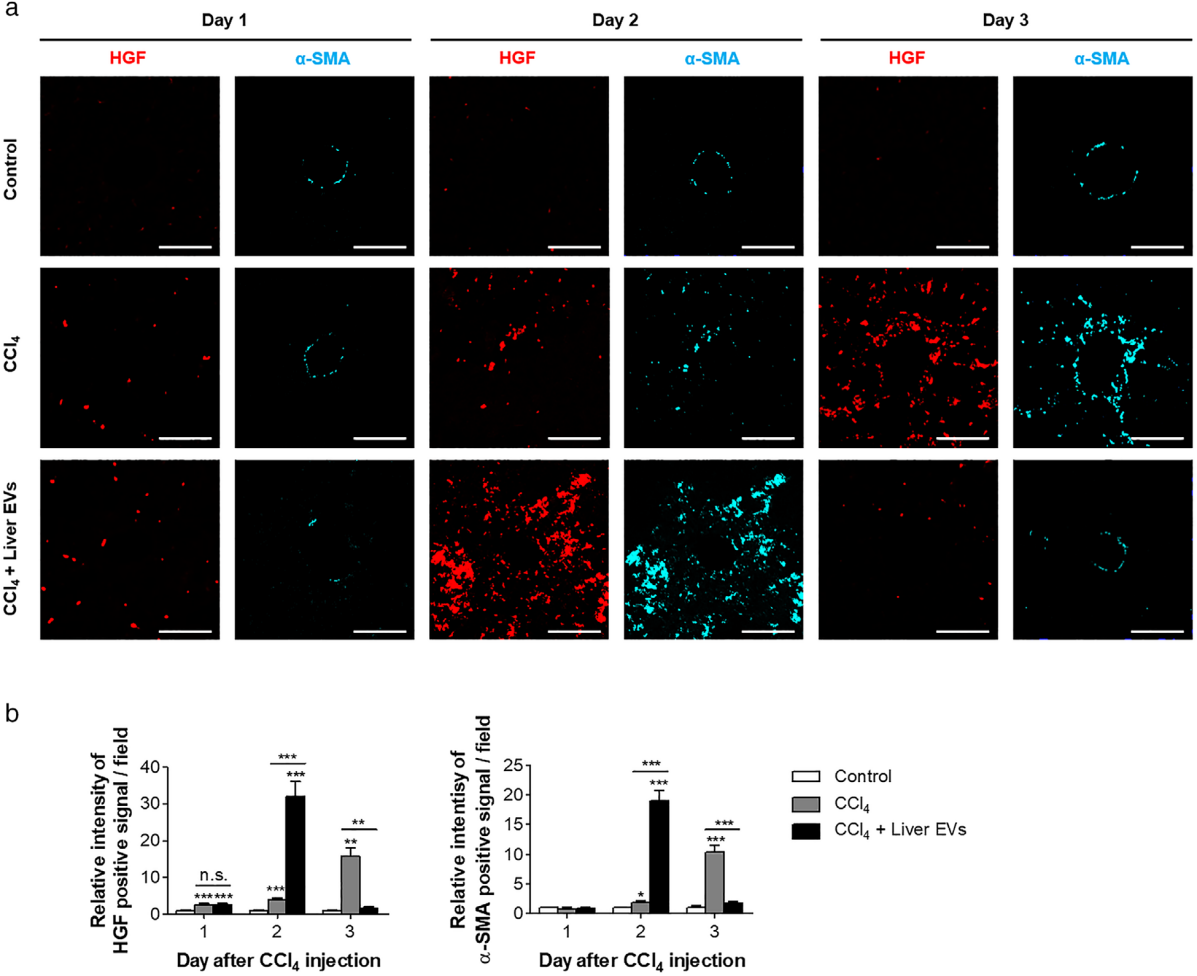
Upregulation of HGF and activated HSCs on damaged area of the liver by in vivo liver EVs. (a). Liver sections were stained with markers for HGF (anti‐HGF antibody; red) and activated HSCs (anti‐α‐SMA antibody; cyan), and signals were detected using a confocal microscope. (b). The relative intensities of the signal were calculated in 10 randomly selected fields per image. Scale bars: 50 μm. Data are presented as the mean ± SD. n.s., not significant; * P < 0.05; ** P < 0.01; *** P < 0.001

Based on the results above, we next investigated the mechanism associated with the proliferative effect of in vivo liver EVs. According to previous literatures, HGF is a major mitogenic factor for hepatocyte proliferation and survival (Bilzer et al., 2006; Friedman, 2008; Kordes et al., 2014; Nakamura & Mizuno, 2010; Taub, 2004; Xiao et al., 2001; Yin et al., 2013). HGF are mostly produced by HSCs, liver‐specific mesenchymal stem cells, quiescent in healthy liver (Kordes et al., 2014; Yin et al., 2013). Upon hepatic injuries by toxins such as CCl_4_, quiescent HSCs are activated by Kupffer cells (Bilzer et al., 2006; Friedman, 2008) and secrete HGF which promotes hepatocyte proliferation. As shown in Figure 6, signal for α‐SMA, a marker for activated HSCs was increased at day 3 of post‐CCl_4_ treatment in the group treated with CCl_4_ alone. In addition, HGF signal also accumulated with the α‐SMA signal. Unlike the group treated with only CCl_4_, the group receiving both CCl_4_ and in vivo liver EVs showed significantly elevated signals of HGF and α‐SMA at day 2 of post‐CCl_4_ treatment which rapidly subsided to the level similar to the negative control group not injected with CCl_4_. Similarly, the level of Kupffer cells, which are a type of macrophages in the liver known to activate HSCs, was also elevated at day 2 of post‐CCl_4_ treatment (Figure S2). These results demonstrate that in vivo liver EVs facilitate the activation of HSCs and their HGF secretion for the recovery of damaged tissues. Collectively, we found that the treatment of in vivo liver EVs to damaged liver tissues accelerates and stimulates the upregulation of HGF, thus expediting the spontaneous recovery of liver tissues. In addition, the concomitant upregulation of activated HSCs and Kupffer cells at the site of tissue damages suggests that the in vivo liver EVs play important roles in the liver regeneration process by activating HSCs.

### 3.6 Treatment of CCl_4_‐induced damaged liver EVs attenuate hepatic injury caused by CCl_4_

To examine the effect of in vivo liver EVs harvested under pathological condition, we isolated EVs from CCl_4_‐induced damaged liver tissue and examined their effect on liver injury caused by CCl_4_. Damaged liver tissues were collected from mice after 48 h of CCl_4_ (2 mg/kg) intraperitoneal administration and EVs were isolated from these damaged liver tissues (Figures S3a and S4). We first performed characterization experiments to determine the biophysical and biochemical features of the purified in vivo CCl_4_‐induced damaged liver EVs and compared with in vivo normal liver EVs. DLS measurement revealed that the average distribution of in vivo CCl_4_‐induced damaged liver EVs is 87.2 ± 5.2 nm and electron microscopic analyses showed CCl_4_ ‐induced damaged liver EVs have homologous morphology with lipid‐bilayered structures (Figure S3b and S3c). In addition, Western blot analyses showed that both normal liver EVs and CCl_4_ ‐induced damaged liver EVs had similar high intensity for EV marker proteins CD9 and CD81 but lacked intracellular organelles GM130 and histone H2B (Figure S3d and S4). Moreover, CCl_4_‐induced damaged liver EVs had EV productivity of 0.70 (± 0.41) x 10^11^ particles from 1 g of CCl_4_‐damaged liver tissues, which is similar to the EV productivity of normal liver EVs which is 0.77 (± 0.16)  × 10^11^ particles from 1 g of normal liver tissues.

**FIGURE 7:**
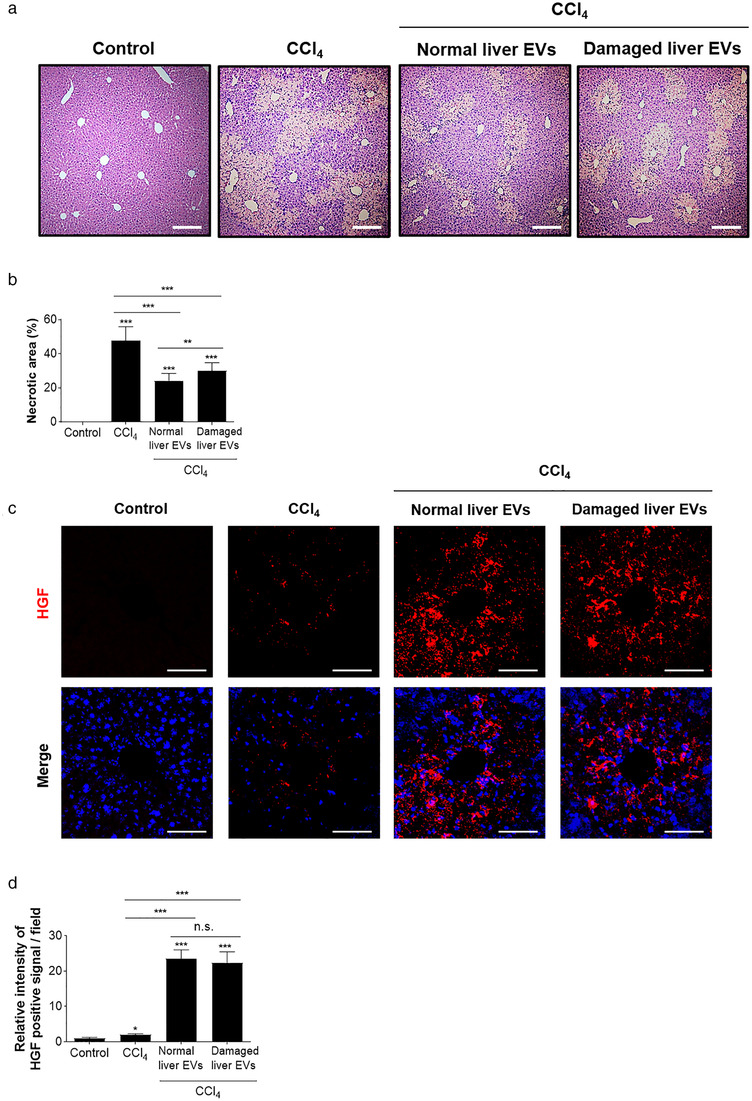
Effect of CCl4‐induced damaged liver tissue‐derived EVs on liver injury. (a). Haematoxylin and eosin staining of liver sections after CCl4 injection. Scale bars: 200 μm. (b). The necrotic areas were calculated in 10 randomly selected fields per section. (c). Liver sections were stained with marker for HGF (anti‐HGF antibody; red) and nucleus (Hoechst; blue) and were detected using a confocal microscope. (d). The relative intensities of the signal were calculated in 10 randomly selected fields per image. Scale bars: 50 μm. Data are presented as the mean ± SD. n.s., not significant; * P < 0.05; *** P < 0.001. ‘Normal liver EVs’ represent for EVs isolated from normal liver and ‘Damaged liver EVs’ represents for EVs isolated from CCl4‐induced damaged liver

Next, we compared the effect of in vivo CCl_4_‐induced damaged liver EVs and in vivo normal liver EVs on the recovery of liver tissue injury due to CCl_4_ treatment. To our surprise, the group treated with in vivo CCl_4_‐induced damaged liver EVs also showed significant degree of attenuated progression of CCl_4_‐induced hepatic necrotic lesions and injury similar to the group treated with in vivo normal liver EVs (Figure 7a and b). In addition, higher HGF signals were accumulated in both groups treated with in vivo normal liver EVs or in vivo CCl_4_‐induced damaged liver EVs, compared to the control group not treated with EVs (Figure 7c and d). Collectively, we found that EVs isolated from CCl_4_‐induced damaged liver tissue have preserved ability to significantly lessen the liver injuries and accelerate liver tissue recovery through accumulation of HGF signal. These results suggest that both normal liver EVs and damaged liver EVs play important pathophysiological roles in maintaining homeostasis after tissue damage. Together, our findings provide new insight into developing in vivo EV‐based therapeutics for preventing and treating liver diseases.

## 4 DISCUSSION AND CONCLUSION

In this study, we isolated EVs from the liver tissues by minimally disruptive procedures to maintain the integrity of cells comprising the liver since disrupted cells during tissue processing could contaminate EVs with cellular dusts and subcellular organelles. Once contaminated, cellular dusts and subcellular organelles are difficult to exclude using the current EV isolation methods. We therefore combined previous EV preparation methods to establish a new *in vivo* liver EV isolation protocol in order to effectively purify large quantity of EVs directly from dissected liver tissues. The in vivo liver EVs were free of common contaminants found in many EV preparations isolated using the previous single‐step EV isolation methods. Moreover, our in vivo liver EVs exhibited typical physicochemical features of EVs including enrichment of tetraspanins and having the lipid bilayer spherical structure and size of around 100 nm in diameter.

From the idea that EVs present in extracellular spaces of the liver tissues are mostly secreted from hepatocytes and regulatory non‐parenchymal cells, *in vivo* liver EVs are expected to exert important roles in maintaining the homeostasis of functional and structural integrities of the liver. Although number of previous studies have shown that cell‐derived EVs isolated from cultured hepatocytes or stem cells have the ability to accelerate liver tissue repair and proliferation, no study was done using in vivo liver EVs to investigate their role in pathophysiological condition. Thus, in order to understand the pathophysiological role and the therapeutic efficacy of in vivo liver EVs on the recovery of liver tissue damaged by CCl_4_ in mice, we isolated in vivo liver EVs from both normal and CCl_4_‐induced damaged liver tissue. We here verified that exogenous administration of EVs isolated from the normal liver tissues accelerates the recovery of necrotic lesions in damaged liver from CCl_4_ treatment, which subsequently resulted in a rapid decrease of the elevated blood levels of ALT, AST and LDH. To our surprise, this protective effect of liver EVs was preserved in EVs isolated from CCl_4_‐induced damaged liver tissue. These results suggest that EVs isolated from both normal liver tissue and damaged liver tissue play important roles in maintaining homoeostasis after tissue damage. In addition, we newly discovered that the treatment of *in vivo* liver EVs attenuates the progression of apoptotic pathway induced by CCl_4_ and reciprocally induces the proliferation of hepatocytes. This implies that this attenuated liver injury could be through the elevated tissue level of HGF secreted from activated HSCs. However, to fully verify the mechanism behind liver regeneration and proliferation via EVs, further studies on vesicular component profiling including multi‐omics studies to identify the vesicular proteins, mRNAs and miRNAs, are needed. These further studies could also be expanded to explain why normal liver EVs isolated from physiological condition and damaged liver EVs isolated from pathologic condition have similar ability of restoring tissue damage.

Although additional studies regarding compositional analysis to classify the sources of *in vivo* EVs and distribution pattern of in vivo liver EVs in the body upon intravenous administration are required, we speculate that most of the in vivo liver EVs injected would localize in the liver since majority of the intravenously injected molecules are targeted to the liver for metabolic modification. Therefore, in vivo liver EVs might have a great potential as a new source of novel therapeutics for preventing and treating liver injuries that could lead to acute and chronic liver failures. In conclusion, our study collectively suggests that further research in the future could possibly allow the development of novel technology that utilize in vivo liver EVs harvested from liver tissue graft of patient with liver injury to aid liver regeneration process without the need for a healthy donor although several significant challenges such as achieving good manufacturing practice (GMP) remain.

## CONFLICT OF INTEREST

The authors report no conflicts of interest.

## Supporting information

Supporting Information

Supporting Information

Supporting Information

Supporting Information

Supporting Information

Supporting Information
