# Supplementary material for: Extracellular vesicles from in vivo liver tissue accelerate recovery of liver necrosis induced by carbon tetrachloride
Source: J Extracell Vesicles. 2021 Aug 11;10(10):e12133. doi: 10.1002/jev2.12133 (PMC8357636; doi:10.1002/jev2.12133)
Supplement: Supplementary file 1 — Supporting Information [file JEV2-10-e12133-s002.docx]

**Extracellular vesicles from *in vivo* liver tissue accelerate recovery of liver necrosis induced by carbon tetrachloride**

Jaemin Lee^1,*^, Sae Rom Kim^1,*^, Changjin Lee^1,*^, Ye In Jun^1^, Seoyoon Bae^1^, Yae Jin Yoon^2^, Oh Youn Kim^1,3,#^ and Yong Song Gho^1,#^

^1^ Department of Life Sciences, Pohang University of Science and Technology (POSTECH), Pohang 37673, Republic of Korea

^2^ Genome Editing Research Center, Korea Research Institute of Bioscience and Biotechnology, Daejeon 34141, Republic of Korea

^3^ Yonsei University College of Medicine, Seoul 03722, Republic of Korea

**^#^Correspondence:**

Yong Song Gho

Department of Life Sciences, Pohang University of Science and Technology (POSTECH), 77 Cheongam-ro, Pohang 37673, Republic of Korea

(E-mail) ysgho@postech.ac.kr

Oh Youn Kim

Department of Life Sciences, Pohang University of Science and Technology (POSTECH), 77 Cheongam-ro, Pohang 37673, Republic of Korea; Yonsei University College of Medicine, Seoul 03722, Republic of Korea

(E-mail) aglaia@postech.ac.kr

^*^ These authors contributed equally to this work.

**Supplementary Figures**


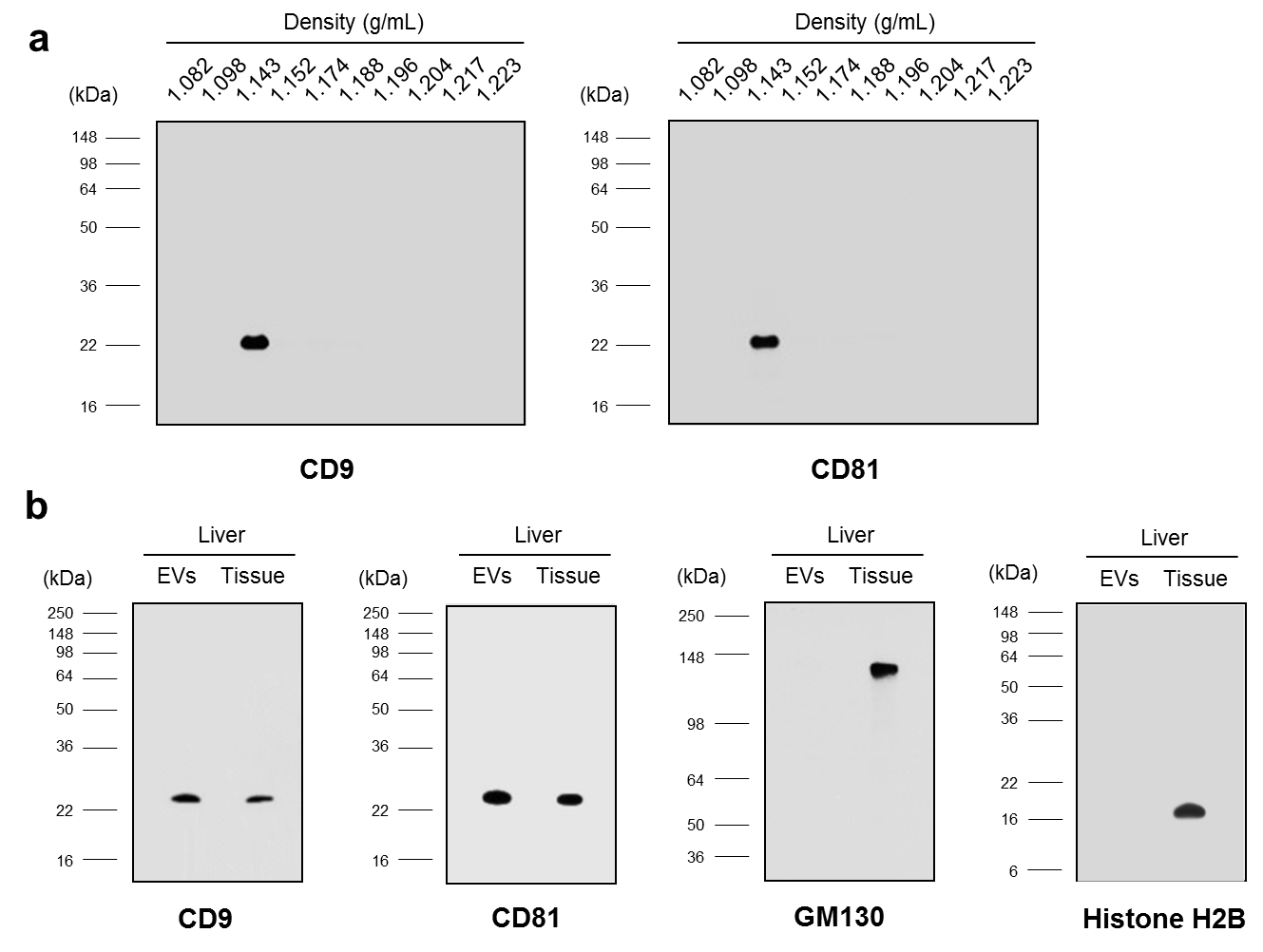


**Figure S1. Uncropped images of western blotting analysis of Figure 1.** a. Western blots of EV markers (CD9 and CD81) in fractions obtained from the iodixanol density gradients of *in vivo* liver EVs. b. Western blots of EV markers (CD9 and CD81), Golgi marker (GM130), and nucleus marker (Histone H2B) of *in vivo* liver EVs and liver lysates.


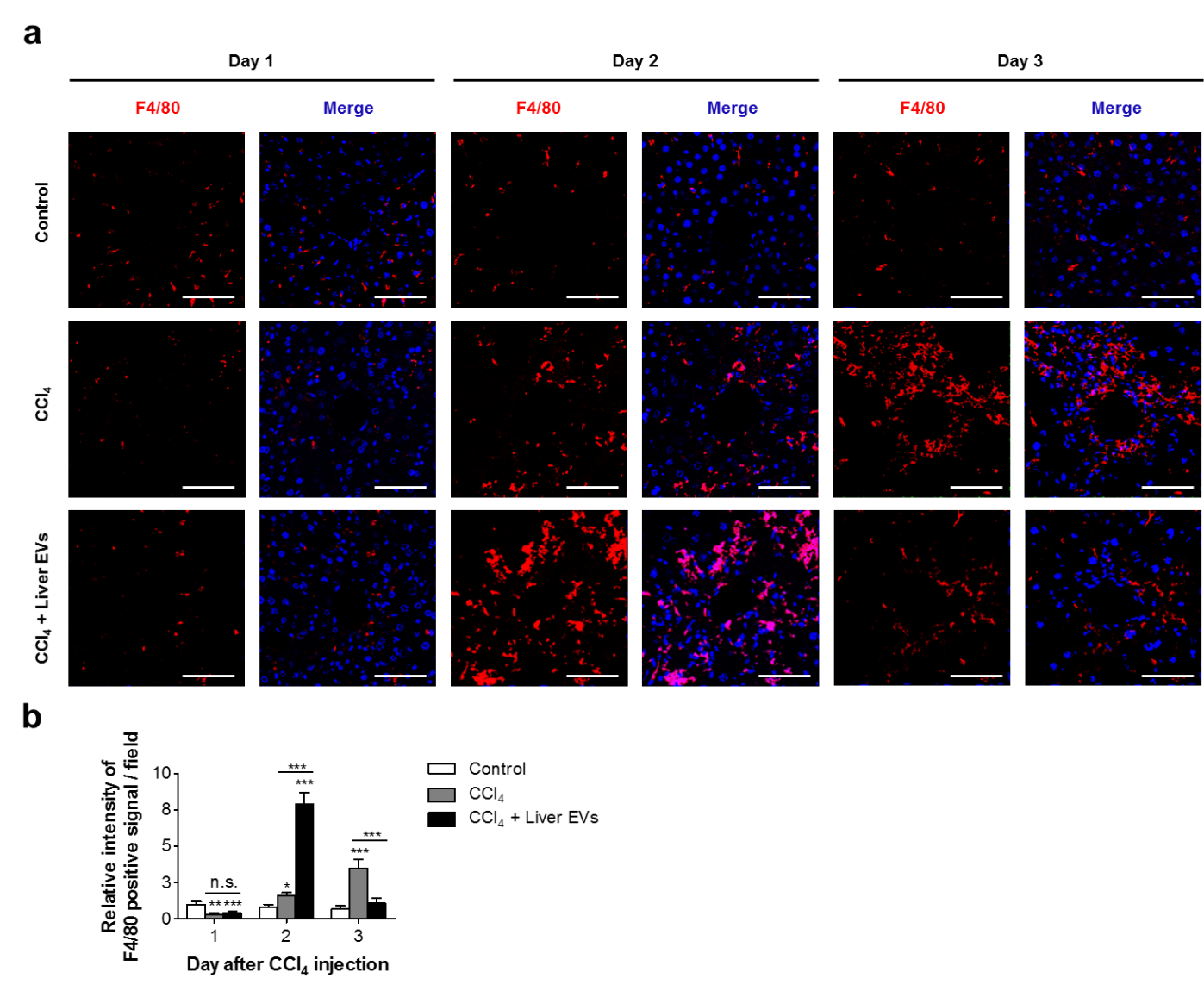


**Figure S2. Infiltration of Kupffer cells on damaged area.** a. The liver sections were stained with markers for Kupffer cells (anti-F4/80 antibody; red) and nucleus (Hoechst; blue), and detected using a confocal microscope. b. The relative intensities of signal were calculated in 10 randomly selected fields per image. Scale bars: 50 μm. Data are presented as the mean ± SD. *n.s.,* not significant; * *P* < 0.05; ** *P* < 0.01; *** *P* < 0.001.


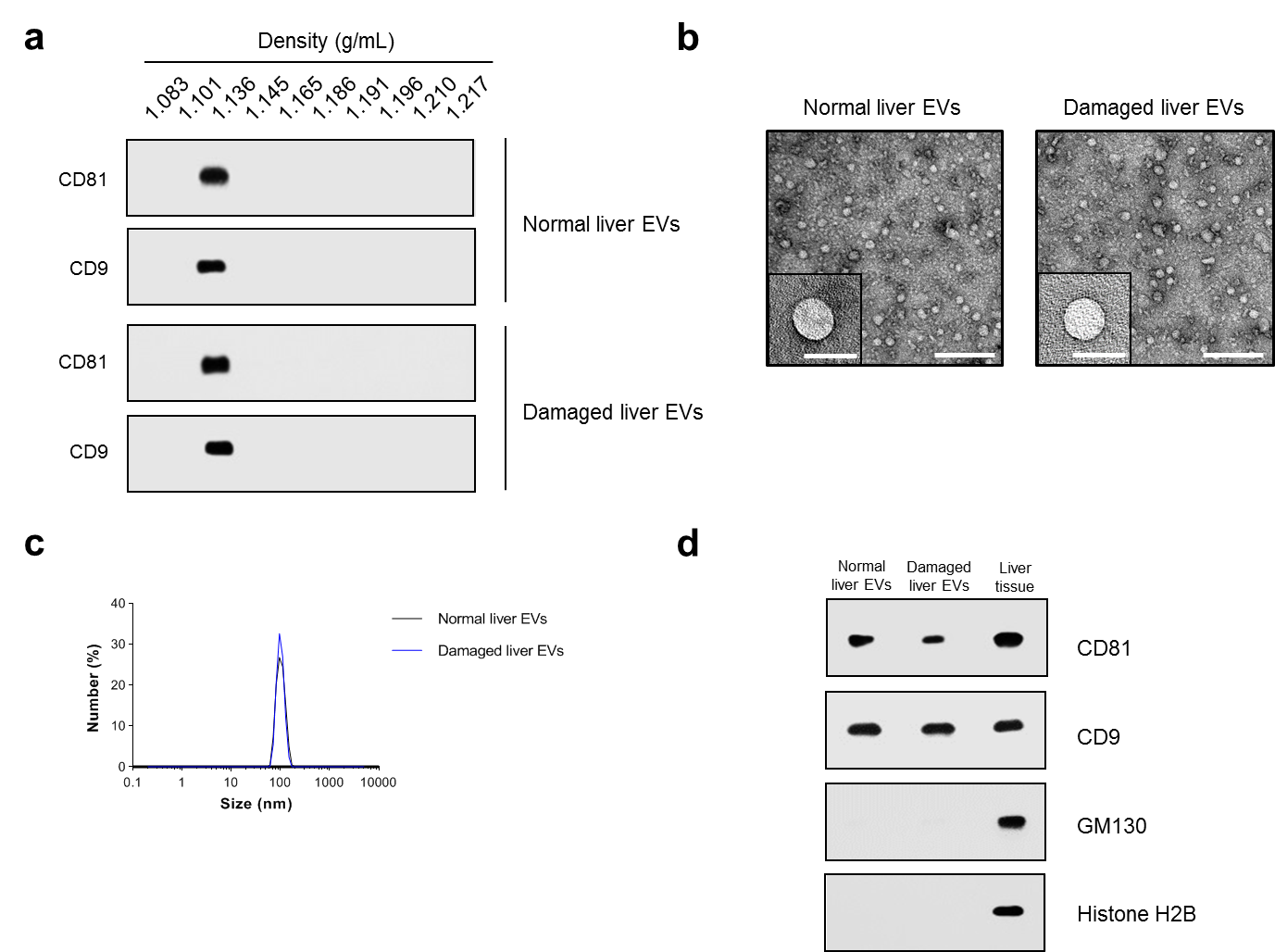


**Figure S3. Characterization of EVs isolated from CCl_4_-induced damaged liver.** a. Western blotting analysis of EV markers, CD9 and CD81, in fractions obtained from the iodixanol density gradients of *in vivo* normal and damaged liver EVs. b. Representative TEM image of *in vivo* liver EVs. Scale bar: 500 nm; inset, 100 nm. c. Size distribution of *in vivo* normal and damaged liver EVs measured by dynamic light scattering (n=3). d. The purified *in vivo* normal and damaged liver EVs (1 μg of total protein) and liver lysates (10 μg of total protein) were loaded to detect the EV markers, CD9 and CD81, and non-EV markers, GM130 and Histone H2B *via* western blotting analysis. “Normal liver EVs” represent for EVs isolated from normal liver and “Damaged liver EVs” represents for EVs isolated from CCl_4_-induced damaged liver.


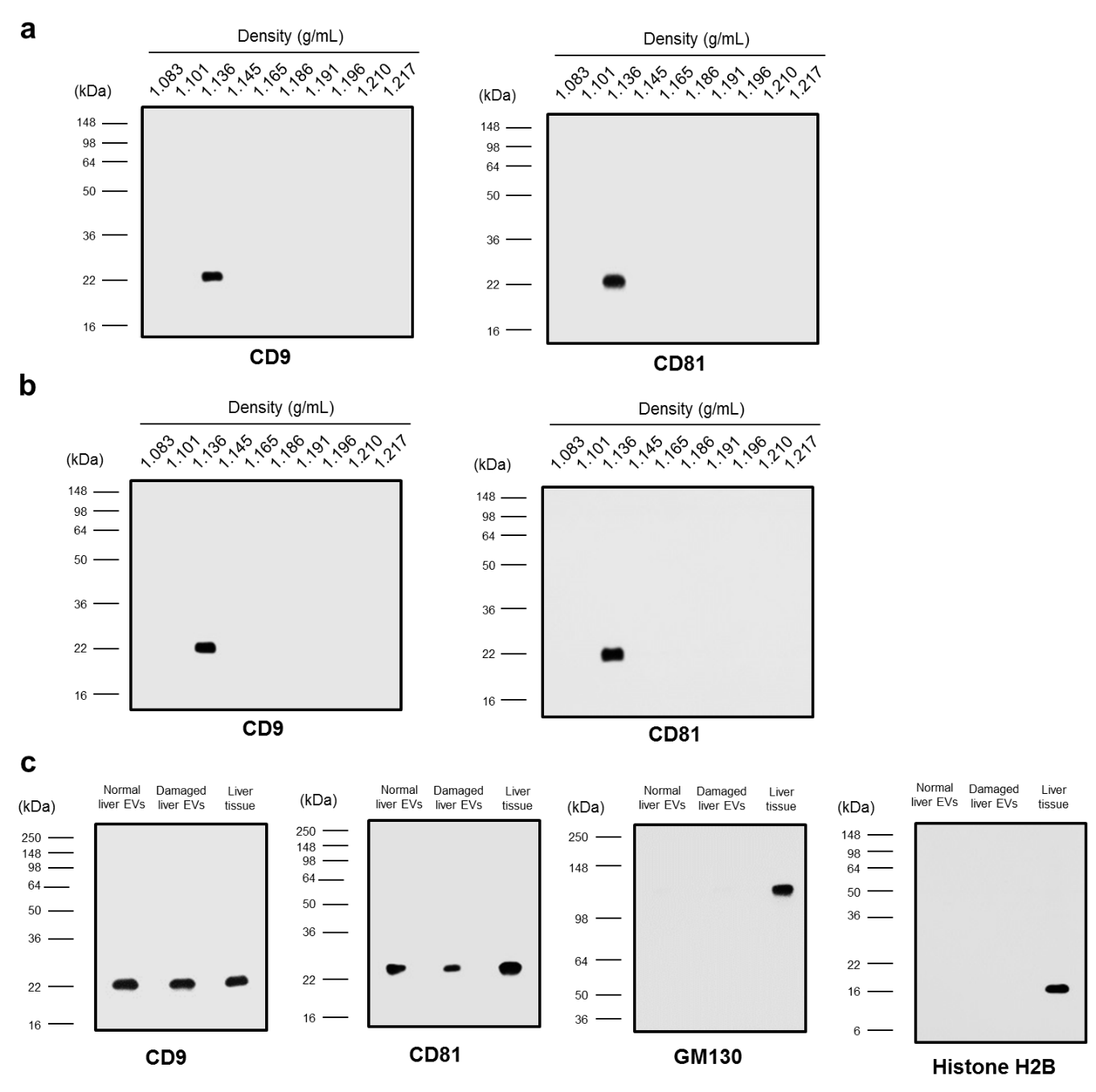


**Figure S4. Uncropped images of western blotting analysis of Figure S3.** a. Western blots of EV markers (CD9 and CD81) in fractions obtained from the iodixanol density gradients of *in vivo* normal liver EVs. a. Western blots of EV markers (CD9 and CD81) in fractions obtained from the iodixanol density gradients of *in vivo* damaged liver EVs. c. Western blots of EV markers (CD9 and CD81), Golgi marker (GM130), and nucleus marker (Histone H2B) of *in vivo* normal and damaged liver EVs and liver lysates. “Normal liver EVs” represent for EVs isolated from normal liver and “Damaged liver EVs” represents for EVs isolated from CCl_4_-induced damaged liver.
