# Supplementary figures and images for: Extracellular vesicles from in vivo liver tissue accelerate recovery of liver necrosis induced by carbon tetrachloride
Source: J Extracell Vesicles. 2021 Aug 11;10(10):e12133. doi: 10.1002/jev2.12133 (PMC8357636; doi:10.1002/jev2.12133)

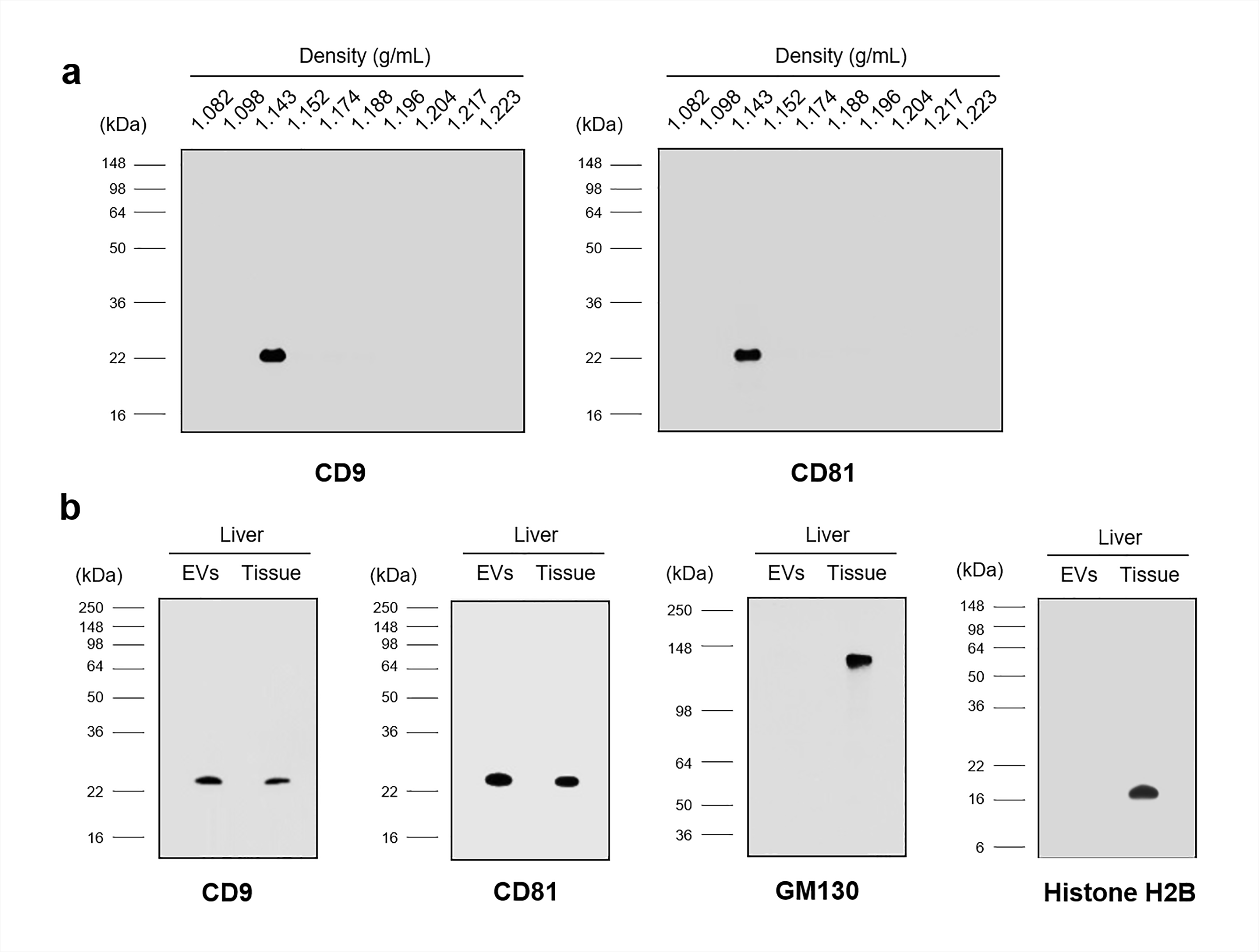

Supplement: Supplementary file 3 — Supporting Information [file JEV2-10-e12133-s003.tif]

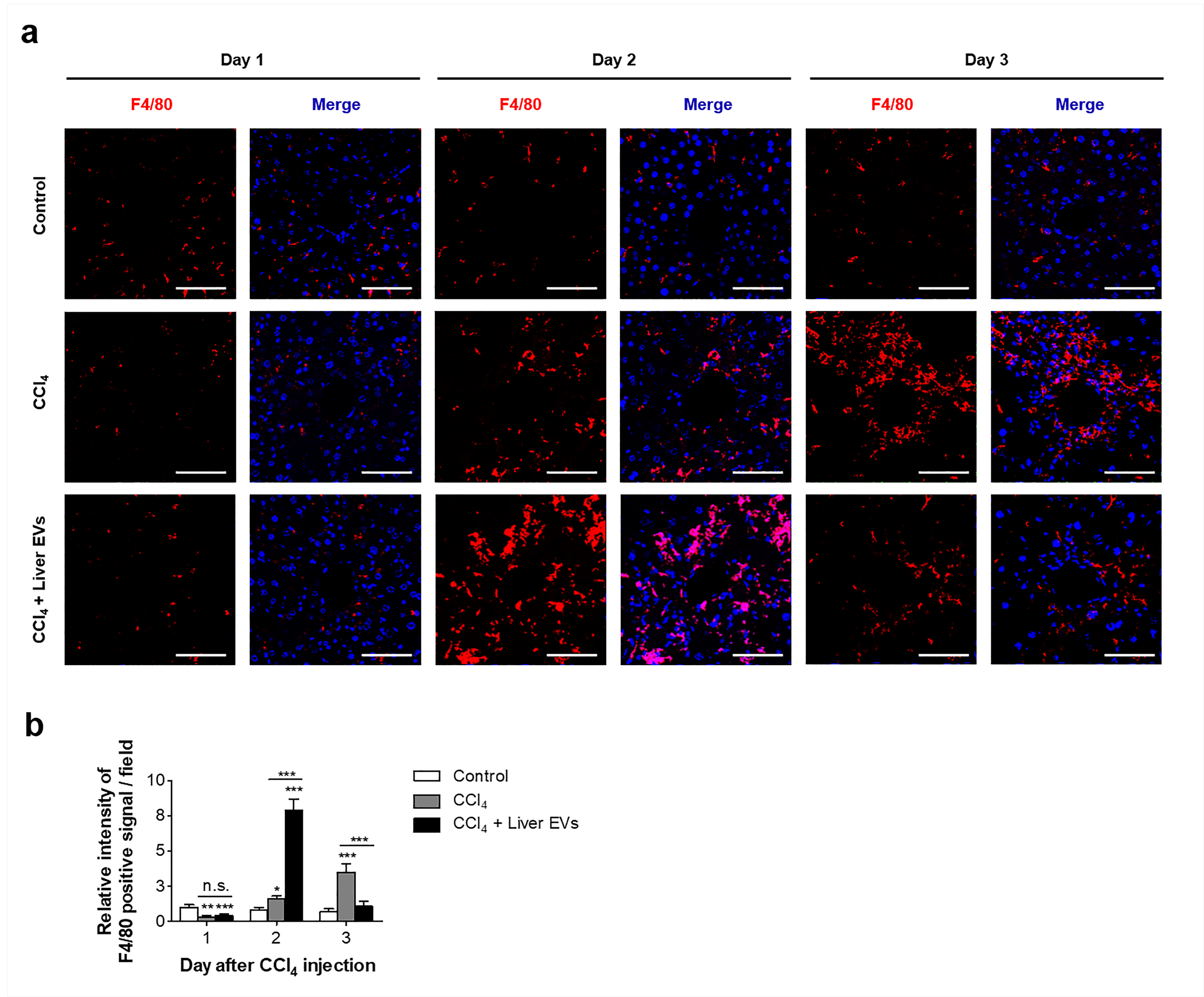

Supplement: Supplementary file 4 — Supporting Information [file JEV2-10-e12133-s005.tif]

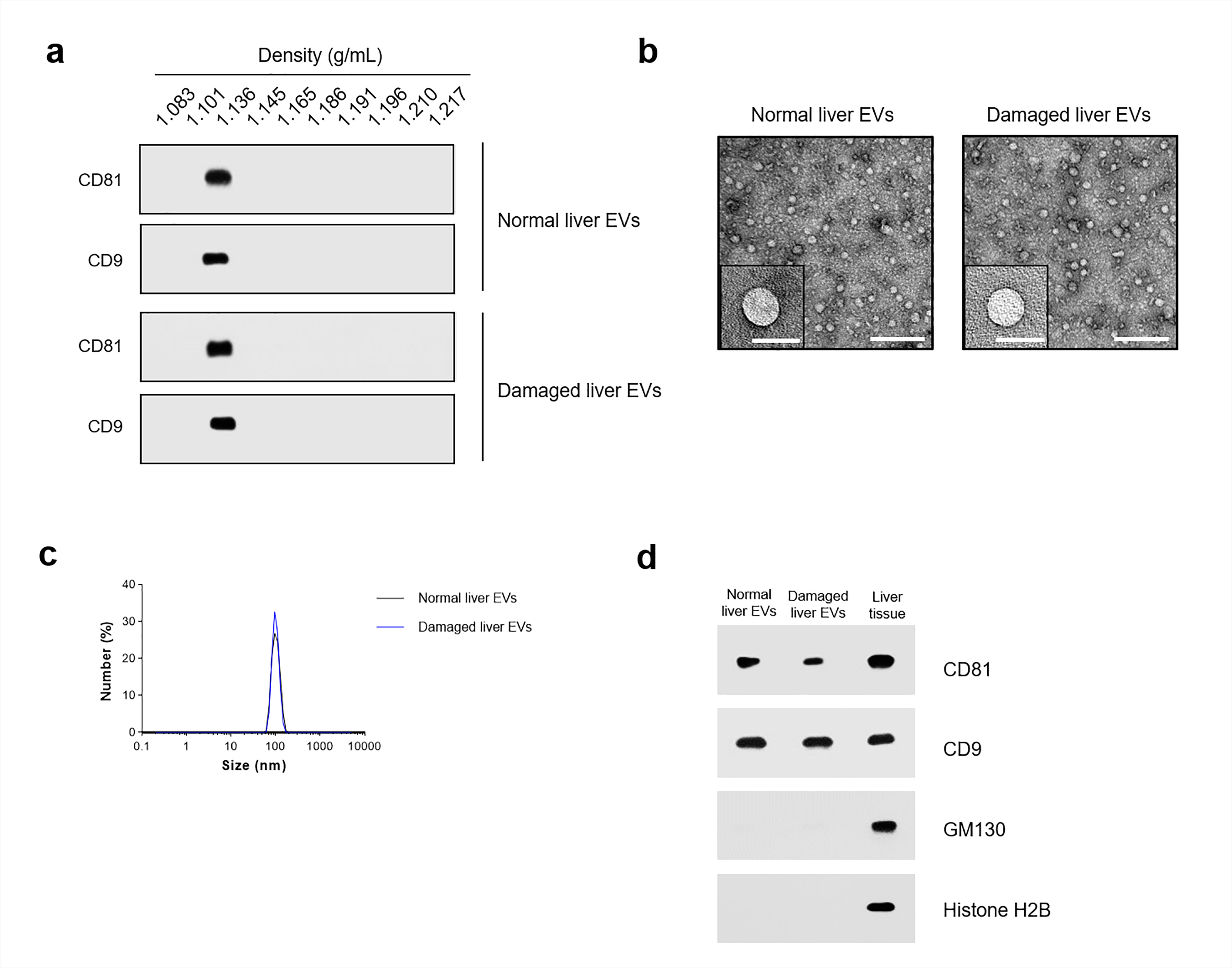

Supplement: Supplementary file 5 — Supporting Information [file JEV2-10-e12133-s001.tif]

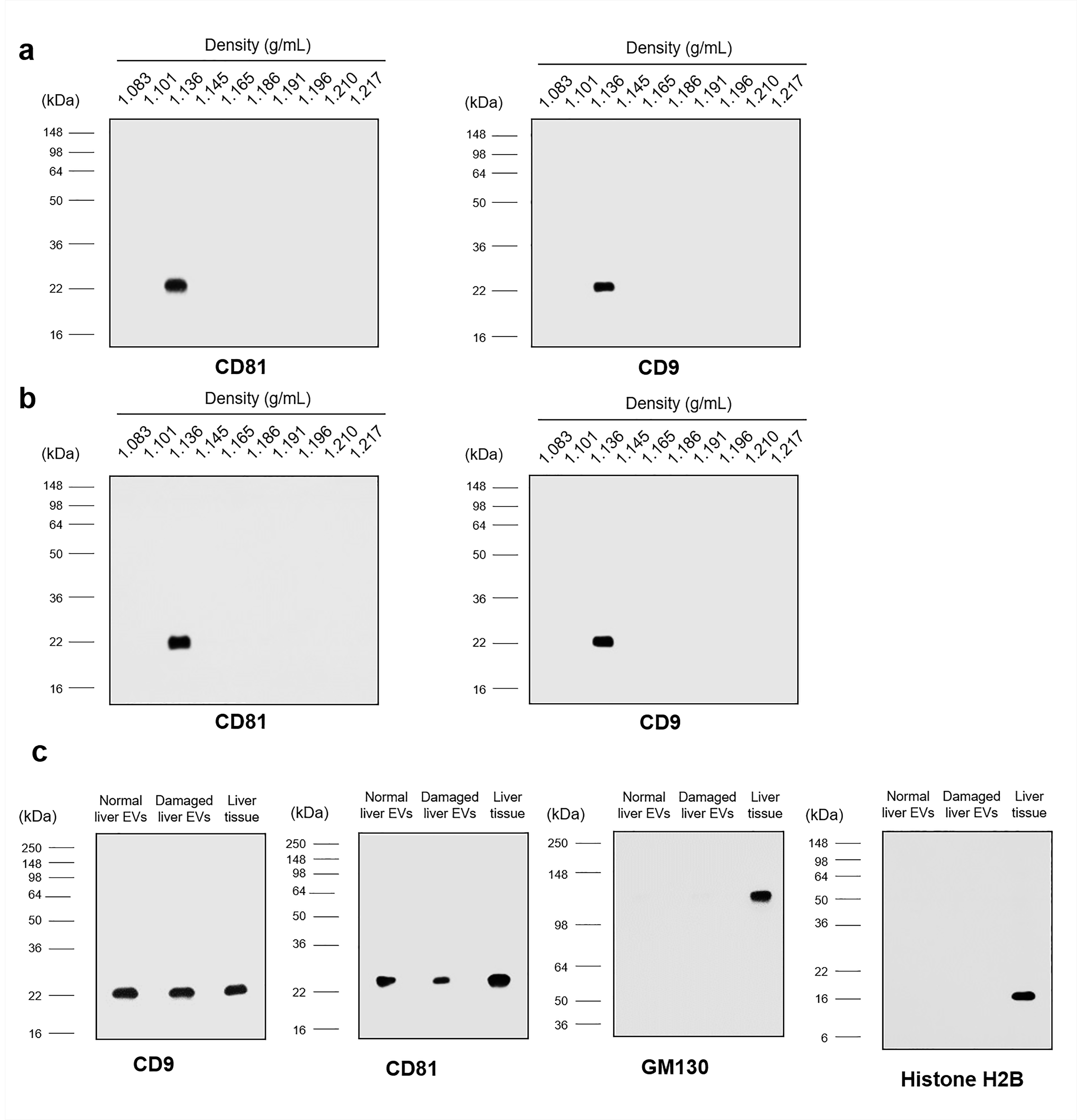

Supplement: Supplementary file 6 — Supporting Information [file JEV2-10-e12133-s006.tif]
